# Model and Data-Driven Combination: A Fault Diagnosis and Localization Method for Unknown Fault Size of Quadrotor UAV Actuator Based on Extended State Observer and Deep Forest

**DOI:** 10.3390/s22197355

**Published:** 2022-09-28

**Authors:** Jia Song, Weize Shang, Shaojie Ai, Kai Zhao

**Affiliations:** School of Astronautics, Beihang University, Beijing 100191, China

**Keywords:** deep forest, extended state observer, fault diagnosis, quadrotor UAV, data-driven

## Abstract

The rotor is an essential actuator of quadrotor UAV, and is prone to failure due to high speed rotation and environmental disturbances. It is difficult to diagnose rotor faults and identify the fault localization simultaneously. In this paper, we propose a fault diagnosis and localization scheme based on the Extended State Observer (ESO) and Deep Forest (DF). This scheme can accurately complete the fault diagnosis and localization for the quadrotor UAV actuator without knowing the fault size by combining the model-based and the data-driven methods. First, we obtain the angular acceleration residual signal of the quadrotor UAV by using ESO. The residual signal is the difference between the observed state of ESO and the true fault state. Then, we design the residual feature analysis method by considering the position distribution of the quadrotor UAV actuator. This method can embed the actuator fault localization information into the fault data by simultaneously considering pitch and roll of the quadrotor UAV. Finally, we complete the fault diagnosis and localization of the quadrotor UAV actuator by processing the fault data by using DF. This scheme has the advantages of straightforward observer modeling, strong generalization ability, adaptability to small sample data, and few hyperparameters. Our simulation results indicate that the accuracy of the proposed scheme reaches more than 99% for the unknown size of the quadrotor UAV actuator fault.

## 1. Introduction

In recent years, quadrotor Unmanned Aerial Vehicle (UAV) has gradually become an important part of people’s lives [1]. The quadrotor UAV has the advantages of simple operation, vertical take-off and landing, and good flexibility [2]. It is widely used in video shooting, agricultural cultivation, logistics transportation, and other fields [3]. With the research on the reliability of quadrotor UAV, the fault diagnosis of quadrotor UAV actuator has also become one of the research hotspots [4]. The actuator of quadrotor UAV is usually in the working state of high speed rotation [5]. Due to frequent task execution and environmental interference, these actuators usually experience various failures leading to efficiency reduction, circuit, and mechanical damage [6]. Accurately finding the actuator fault localization and determining the fault type can greatly improve the reliability techniques of quadrotor UAV [7]. Therefore, it is necessary to study the fault diagnosis for the quadrotor UAV actuator [8].

The fault diagnosis methods for the quadrotor UAV actuator are mainly categorized into model-based and data-driven methods [9]. Ren et al. [10] completed the fault diagnosis of quadrotor UAV actuator by designing the Robust H-Infinity Observer. Gao et al. [11] combined Extended Kalman Filter and adaptive estimation method to diagnose actuator fault types. The above model-based methods require accurate mathematical modeling of the quadrotor UAV and the actuator [12]. Model-based methods are also difficult to effectively achieve accurate modeling of interference and various errors. With the development of machine learning technique, data-driven methods are widely used in fault diagnosis [13]. Park et al. [14] applied the neural network method to the actuator fault diagnosis of a quadrotor UAV under unknown control signals. In this method, the network with hundreds of layers increases the computation amount and the design process is complicated. Fu et al. [9] combined convolutional neural networks and long short-term memory networks to establish the fault localization model of UAV actuator. Although this method completed the fault localization of the actuator, it did not consider the fault diagnosis of different types of actuator faults. The data-driven method can achieve high fault diagnosis accuracy without relying on the model, but it is prone to overfitting and cannot effectively deal with the fault diagnosis under the condition of system state change.

Actuator fault type diagnosis and fault localization identification are very important for quadrotor UAV actuator fault diagnosis, but they are usually carried out separately. In this paper, we propose a fault diagnosis and localization scheme for a quadrotor UAV actuator based on ESO-DF under the condition of unknown fault size. In this scheme, model-based and data-driven methods are combined to realize fault type diagnosis and fault localization identification simultaneously. The Extended State Observer (ESO) is an observer in Active Disturbance Rejection Control (ADRC) [15]. ESO can track the motion state of the system quickly and accurately with a small number of parameters [16]. Li et al. [17] has achieved good results by applying ESO to autonomous underwater vehicle actuator fault diagnosis. DF is an ensemble learning method based on decision tree proposed by Zhou et al. [18]. This method has the advantages of less hyperparameters, less computation, and small sample size. Qin et al. [19] applied DF to realize the fault diagnosis of rolling bearing. For the fault diagnosis of quadrotor UAV, Ai et al. [20] applied DF to the fault diagnosis of quadrotor UAV for the first time, which verifies the effectiveness of DF in fault diagnosis.

In order to better extract the fault features of quadrotor UAV actuators, we have made corresponding improvements to ESO [21]. We extract the angular acceleration of pitch and roll through the improved ESO to extract fault features. In addition, in order to confirm the location of the actuator failure, we designed an actuator residual feature analysis method combined with the location distribution of the quadrotor UAV actuator [22]. Through this method, we can embed the fault location information of the actuator into the residual fault data. Finally, we completed the fault type diagnosis and fault localization identification of the quadrotor UAV actuator by using the DF method.

The contributions of this paper are summarized as follows:

*1.*  *We have improved the ESO combined with the characteristics of the quadrotor UAV actuator failure. We obtain the angular acceleration containing the fault characteristics of the quadrotor UAV actuator through ESO.*
*2.*  *Aiming at the problem of fault location identification of quadrotor UAV actuators, we designed an analysis method of actuator fault residual characteristics. By combining the angular acceleration information of pitch and roll, the method embeds the fault location information of the actuator into the fault data.*
*3.*  *Aiming at the problem that the judgment of the fault type and the fault location cannot be obtained at the same time, we designed an actuator fault diagnosis and localization scheme based on ESO-DF. By combining the model-based method and the data-driven method, the scheme fully extracts fault features and achieves high-accuracy fault diagnosis and localization results.*

The remainder of this paper is organized as follows. The problem definition and basic theory are introduced in Section 2. In Section 3, the proposed method is introduced. In Section 4, the setup of the simulation experiments is presented and the results of the experiments are analyzed. Finally, conclusion is given in Section 5.

## 2. Preliminaries

### 2.1. Mathematical Model of Quadrotor UAV

**Figure 1:**
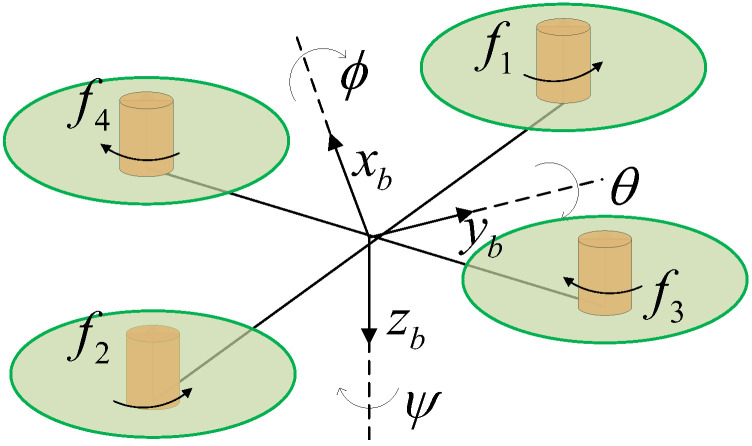
The body fix reference frame of the quadrotor UAV. The $x_{b}$ axis is set to be parallel to the body and points toward the nose. The $y_{b}$ axis is set to be perpendicular to the body and points to the right. The $z_{b}$ axis is set to be perpendicular to the $x_{b}$–$y_{b}$ plane and pointing downwards.

In the simulation experiment of this paper, we have the following three assumptions on the mathematical model of the quadrotor UAV:

- The quadrotor UAV is a rigid body.
- The mass of the quadrotor UAV is uniformly distributed and the center of mass overlaps with the geometric center.
- The four actuators of the quadrotor UAV are evenly distributed on the fuselage according to the X shape, as shown in Figure 1.

The six-degree-of-freedom mathematical model [23] of the quadrotor UAV is shown below:

(1) $$\ddot{\phi }=\left(\frac{J_{y}-J_{z}}{J_{x}}\right)\dot{\theta }\dot{\psi }+\frac{j_{r}}{J_{x}}\dot{\theta }\left(\begin{array}{c} -\omega_{1}+\omega_{2}-\omega_{3}+\omega_{4} \end{array}\right)+\frac{1}{J_{x}}U_{2}$$

(2) $$\ddot{\theta }=\left(\frac{J_{z}-J_{x}}{J_{y}}\right)\dot{\phi }\dot{\psi }-\frac{j_{r}}{J_{y}}\dot{\phi }\left(\begin{array}{c} -\omega_{1}+\omega_{2}-\omega_{3}+\omega_{4} \end{array}\right)+\frac{1}{J_{y}}U_{3}$$

(3) $$\ddot{\psi }=\left(\frac{J_{x}-J_{y}}{J_{z}}\right)\dot{\phi }\dot{\theta }+\frac{1}{J_{z}}U_{4}$$

(4) $$\ddot{x}=\frac{1}{m}U_{1}\left(\sin\theta \cos\psi \cos\varphi +\sin\psi \sin\varphi \right)-\frac{1}{m}D_{z}{\dot{x}}^{2}$$

(5) $$\ddot{y}=\frac{1}{m}U_{1}\left(\sin\theta \sin\psi \cos\varphi -\cos\psi \sin\varphi \right)-\frac{1}{m}D_{y}{\dot{y}}^{2}$$

(6) $$\ddot{z}=\frac{1}{m}U_{1}\cos\theta \cos\varphi -g-\frac{1}{m}D_{z}{\dot{z}}^{2}$$

where (x,y,z) represents the coordinates of the center of mass of the vehicle under the earth coordinate system. θ, ϕ, and ψ are the pitch, roll and yaw angle, respectively. $j_{r}$ refers to the rotational inertia of the rotor. *m*, *g* represent the mass and gravitational acceleration of the quadrotor drone, respectively. $U_{1}$ represents the lift of a quadrotor UAV. $U_{2}$, $U_{3}$, and $U_{4}$ represent the aerodynamic moment. $D_{x}$, $D_{y}$, and $D_{z}$ are air resistance coefficients of three axes. $J_{x}$, $J_{y}$, and $J_{z}$ refer to the rotational inertia of the body.

### 2.2. Actuator Fault Model

In this paper, the quadrotor UAV actuator faults mainly include speed constant deviation faults and speed gain loss faults [24].

#### 2.2.1. Actuator Speed Constant Deviation Fault

The speed constant deviation fault is usually caused by bias current or bias voltage in the circuit. The fault model is as follows.

(7) $$\omega_{f}\left(t\right)=\left\{\begin{array}{cc} \omega (t) & 0\leq t<t_{f}\\ \omega (t)+\Delta & t_{f}\leq t \end{array}\right.$$

where ω(t) represents the rotational speed of the actuator under no-fault conditions. $\omega_{f}\left(t\right)$ represents the actual rotational speed of the actuator. Δ represents the deviation speed value between the actual value of the actuator rotational speed and the expected value. At $t=t_{f}$, the actuator has a fault with a speed deviation value of Δ.

#### 2.2.2. Actuator Speed Gain Loss Fault

The speed gain loss fault is usually caused by mechanical damage to the motor or propeller. The fault model is as follows.

(8) $$\omega_{f}\left(t\right)=\left\{\begin{array}{cc} \omega (t) & 0\leq t<t_{f}\\ \rho \omega (t) & t_{f}\leq t \end{array}\right.$$

where ω(t) represents the rotational speed of the actuator under no-fault conditions. $\omega_{f}\left(t\right)$ represents the actual rotational speed of the actuator. ρ represents the actuator speed gain loss factor. At $t=t_{f}$, the actuator has a fault with speed gain loss.

### 2.3. Extended State Observer (ESO)

The purpose of ESO is to solve the core problem of the observation of disturbances in ADRC. It borrows the idea of the state observer in modern control theory, that is, on the basis of observing the state variables $a_{1}$, $a_{2}$…$a_{n}$ of the system, all factors that affect the output of the controlled object except the control quantity are called the total disturbance $f\left(x_{1},x_{2},\ldots x_{n}\right)$. Then, it expands the total disturbance into a new state variable $a_{n+1}$, and uses its special feedback mechanism to establish an expanded state observer to observe the total disturbance. The biggest advantage of ESO is that it does not depend on the model that generates the perturbation. It also does not require a detailed and accurate system model to estimate the model parameter uncertainty and the total disturbance value caused by changes in the external environment. In the final output, the observed disturbances are offset in real time to obtain the estimated the total disturbance by ESO.

The following is an introduction to the ESO design of a second-order system. Equation (9) is the ADRC form of a second-order system.

(9) $$\left\{\begin{array}{c} {\dot{x}}_{1}=x_{2}\\ {\dot{x}}_{2}=f\left(x_{1},x_{2},\omega \left(t\right)\right)+bu\\ y=x_{1} \end{array}\right.$$

where $f\left(x_{1},x_{2},\ldots x_{n}\right)$ is the total disturbance. It contains the internal disturbance generated by the system state variable $x_{1}$, $x_{2}$ and the external disturbance ω(t). In order to observe the specific size of $f\left(x_{1},x_{2},\ldots x_{n}\right)$, we introduce a new state variable $x_{3}=f\left(x_{1},x_{2},\omega \left(t\right)\right)$. Substitute $x_{3}=f\left(x_{1},x_{2},\omega \left(t\right)\right)$ into Equation (9) to obtain the expanded second-order system as shown in Equation (10).

(10) $$\left\{\begin{array}{c} {\dot{x}}_{1}=x_{2}\\ {\dot{x}}_{2}=x_{3}+bu\\ {\dot{x}}_{3}=\dot{f}\left(x_{1},x_{2},\omega \left(t\right)\right)\\ y=x_{1} \end{array}\right.$$

For better tracking of the expanded third-order system, the following linear ESO is established for Equation (11):

(11) $$\left\{\begin{array}{c} e_{1}=a_{1}-y\\ {\dot{a}}_{1}=a_{2}-\beta e_{1}\\ {\dot{a}}_{2}=a_{3}-\beta_{2}e_{1}+bu\\ {\dot{a}}_{3}=-\beta_{3}e_{1} \end{array}\right.$$

where $e_{1}$ is the error between the observed quantity $a_{1}$ and the output *y* of the controlled object. *u* is the output value of the controller. $\beta_{1}$, $\beta_{2}$, and $\beta_{3}$ are related to the bandwidth $\omega_{b}$ of the system. when t→∞, $e_{1}\to 0$, in other words, $a_{1}\to x_{1}$, $a_{2}\to x_{2}$, $a_{3}\to x_{3}$. Through the above proof, the observation of the total disturbance can be realized.

### 2.4. Deep Forest

**Figure 2:**
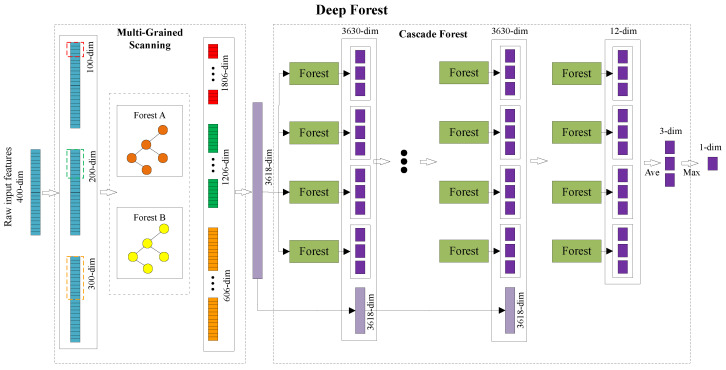
Fundamentals of deep forests. It mainly includes multi-grained scanning and cascade forest.

Deep Forest (DF) is an ensemble learning method based on decision tree proposed by Professor Zhihua Zhou in 2017. DF mainly consists of two parts as shown in Figure 2: Multi-Grained Scanning (MGS) and Cascade Forest (CF). After the original input features are expanded by MGS, they are input into CF for classification. The features are filtered through multiple layers in CF, and the final classification category can be obtained. The following describes the specific implementation process of DF based on the three-category classification of sequences whose original input features are 400 dimensions.

#### 2.4.1. Multi-Grained Scanning

MGS is a method of feature expansion. The original features are cut by sliding windows to enhance the classification effect of CF. In Figure 2, MGS cuts the original 400-dimensional input features through 100-dimensional, 200-dimensional, and 300-dimensional sliding windows with a step size of 1 dimension. In total, 301, 201 and 101 features are obtained, respectively. Then, each segment of the obtained features is input into Forest A and Forest B, respectively. Then, feature extraction is performed on each segment of features through Forest A and Forest B, respectively. After each feature passes through Forest A or Forest B, a three-dimensional feature can be obtained. All the features extracted from Forest A and Forest B obtained by each sliding window are connected together. A 100-dimensional sliding window can obtain 301 (number of features) × 2 (Forest A and Forest B) × 3 (3-dimensional features) = 1806 dimensions, as shown in Figure 2. Similarly, a 200-dimensional sliding window and a 300-dimensional sliding window can obtain 1206-dimensional features and 606-dimensional features, respectively. Finally, by concatenating the features obtained by the three sliding windows, the 3618-dimensional features are obtained as the input of CF.

In MGS, Forest A and Forest B are two kinds of decision trees. Decision trees can complete the classification task of input features. The original 400-dimensional input features are transformed into 3618-dimensional features after feature extraction by MGS. This process completes the enhancement of the original features, which is beneficial to the classification process of CF.

#### 2.4.2. Cascade Forest

CF is a module that completes the classification work inside DF, as shown in Figure 2. CF is composed of multiple layers of forest. Each layer of CF consists of multiple forests. Forest is an optional decision tree classification method, and its number in cascaded forests can also be set. The input to the first layer of CF is 3618-dimensional features obtained by MGS. After each feature passes through each forest in each layer, a 3-dimensional feature can be obtained. Since the default number of forests is 4, the features of 3 × 4 = 12 dimensions can be obtained after each layer. In order to enhance the classification ability, the obtained 12-dimensional features are connected with the input of 3618-dimensional features of the cascade forest and then input to the next layer for processing. CF performs a loss calculation on the classification results of each layer. When the classification results obtained by several consecutive layers do not change, the final classification category can be obtained by calculating the average and maximum values of the classification results of the last layer.

## 3. The Proposed Scheme

In this section, the overall framework of our proposed ESO-DF scheme is presented first in Section 3.1. Then, two key parts of the proposed ESO-DF scheme are described in Section 3.2 and Section 3.3, respectively, namely, the residual acquisition method based on ESO and the designed residual feature analysis method. In addition, Section 3.4 describes the procedure for establishing an actuator fault data set. Finally, the fault diagnosis and localization process of the unknown fault size of the quadrotor UAV actuator based on the proposed ESD-DF scheme is described in detail at the end of this section.

### 3.1. Fault Diagnosis and Localization Scheme Based on ESO-DF

**Figure 3:**
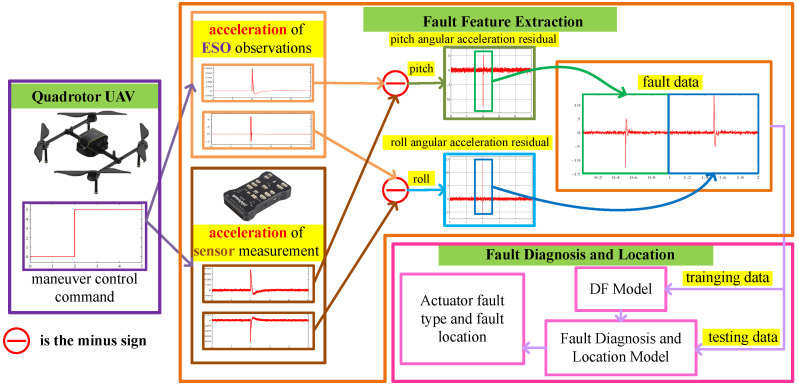
The overall framework of fault diagnosis and localization of the quadrotor UAV actuator based on ESO-DF.

The overall framework of fault diagnosis and localization of the quadrotor UAV actuator based on ESO-DF under the condition of unknown fault size is shown in Figure 3. First, we send maneuver control commands to the quadrotor UAV. The UAV maneuvers according to the control commands. The sensor collects angular acceleration information according to the actual motion state of the UAV. ESO obtains residual data including actuator fault information according to the control torque calculated by the controller and the angular acceleration information collected by the sensor. In order to obtain the actuator fault location information, we designed an actuator residual feature analysis method based on the location distribution of the quadrotor UAV actuator. With this method, the information of the fault location is embedded in the fault data. Finally, a fault data sequence containing fault type and fault location information is obtained. We feed the failure data sequence into the DF for training and testing. Through the above process, we have completed the fault diagnosis and localization of the quadrotor UAV actuator.

### 3.2. Residual Acquisition Method Based on ESO

ESO can accurately track the movement status of the quadrotor UAV according to maneuver control command. ESO can estimate the approximate total disturbance based on the input and output data of the system. The performance of ESO is only related to the estimation accuracy of $b_{0}$ and the observer bandwidth [25]. In Equation (11), the input of the ESO is the control torque and the angle measured by the sensor after the solution, and the output of the ESO is the angle residual information. In order to obtain the fault information of the actuator, we have improved the ESO combined with the fault characteristics of the actuator. When an actuator fails, its failure characteristics will first be reflected in the lift. When there is an abnormal state of lift, the angular acceleration, not the angle, will change significantly. So we made some improvements to ESO based on this feature so that ESO can output angular acceleration residual information. When a certain actuator fails, the angular acceleration residual information output by the ESO will change significantly. Since the pitch and roll channel of quadrotor UAV are similar, the ESO model of angular acceleration of pitch channel is introduced below. The model is as follows:

(12) $$\left.\begin{array}{c} {\dot{a}}_{1}=a_{2}+\beta_{1}e_{\theta }\\ {\dot{a}}_{2}=a_{3}+\beta_{2}e_{\theta }+b_{0}U_{3}\\ {\dot{a}}_{3}=\beta_{3}e_{\theta }\\ e_{\theta }=\theta -a_{1}\\ e_{\ddot{\theta }}=\ddot{\theta }-{\dot{a}}_{2} \end{array}\right\}$$

where $\beta_{1}$, $\beta_{2}$, and $\beta_{3}$ are related to the bandwidth $\omega_{b}$ of the system. $e_{\theta }$ is the residual between the angle observed by the ESO and the angle measured by the sensor. $\ddot{\theta }$ is the measured value of the pitch angle acceleration sensor. $U_{3}$ is the pitch axis control torque, see Equation (2).

When t→∞, $a_{1}\to \theta$, thus $e_{\theta }\to 0$. We take the difference between the ${\dot{a}}_{2}$ observed by the ESO based on the control torque and the angular acceleration $\ddot{\theta }$ measured by the sensor to obtain $e_{\ddot{\theta }}$. $e_{\ddot{\theta }}$ is the residual difference between the angular acceleration observed by ESO and that measured by sensor. From the ESO model of Equation (12), it can be seen that the characteristics of actuator fault under maneuvering conditions can be obtained without introducing additional signals.

### 3.3. Residual Feature Analysis Method

**Figure 4:**
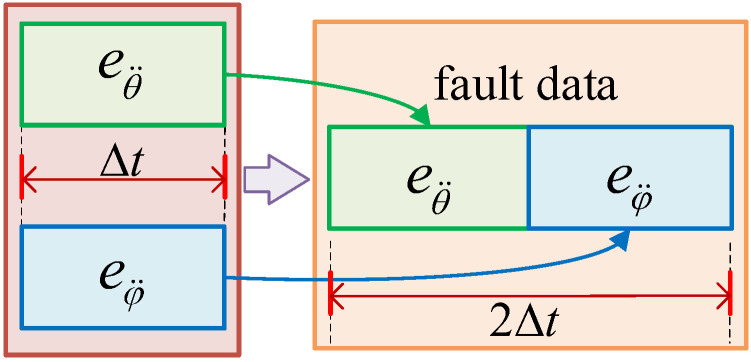
Actuator fault data feature fusion process. Δt is the sampling length. The pitch angular acceleration residuals $e_{\ddot{\theta }}$ and roll angular acceleration residuals $e_{\ddot{\varphi }}$ of the corresponding time are spliced, that is, the fault data containing unknown fault information is finally obtained.

We designed an actuator residual feature analysis method by combining the actuator position distribution of quadrotor UAV. Since the actuators of the quadrotor UAV are X-shaped and evenly distributed around the body, the four actuators will have different effects on pitch and roll when they fail separately; therefore, we embedded fault localization information into the fault data by splicing the angular acceleration of pitch and roll in the time dimension, as shown in Figure 4.

### 3.4. Fault Dataset Establishment

The attitude maneuver of the quadrotor UAV is carried out according to the maneuver control command at $t=t_{d}$. The actuator fails while performing a maneuver, that is, a fault of random size is injected into the actuator at $t=t_{d}$. Then, we obtain angular acceleration residual data by using ESO. After that, the residual signal in $\left[t_{d}-\Delta t/2,t_{d}+\Delta t/2\right]$ is intercepted. We obtain fault data by processing the intercepted residual signal using the actuator residual feature analysis method. Finally, we build the actuator failure dataset by repeating the experiments for N times.

### 3.5. Workflow of Actuator Fault Diagnosis and Localization Based on ESO-DF

**Figure 5:**
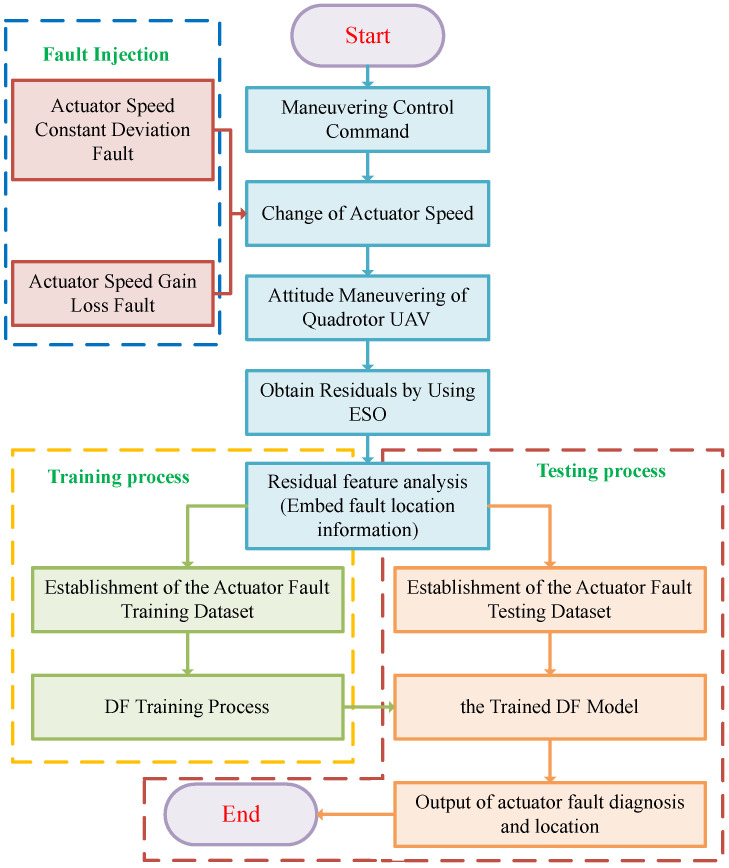
Flow chart of the ESO-DF based actuator fault diagnosis and localization scheme.

The workflow of the proposed ESO-DF based actuator fault diagnosis and localization scheme is depicted in Figure 5.

The entire process can be split into two parts: training and testing. The specific steps are set out below.

#### 3.5.1. Training Process

1. Injecting faults into actuators while maneuvering: injecting faults into actuators while the quadrotor UAV is performing attitude maneuvers.
2. Extracting residual fault signals: According to the control torque and the angular acceleration information measured by the sensor, the ESO outputs the angular acceleration residual sequence containing the actuator fault information, that is, the fault data.
3. Embedding fault localization information into fault data: we apply residual feature analysis method to embed fault localization information into fault data. The fault data obtained by embedding fault location information are a time series of a certain length.
4. Train Fault Diagnosis and Localization Model: we obtain the fault diagnosis and localization model trained by injecting fault data into DF.

#### 3.5.2. Testing Process

1. Obtain testing data: we follow steps 1, 2, and 3 of the training process to obtain test failure data.
2. Actuator fault diagnosis and localization: We complete the fault diagnosis and localization of the quadrotor UAV actuator by processing the test fault data using the trained model.

## 4. Simulation and Results Analysis

### 4.1. Metrics

The evaluation metrics used in this paper include accuracy, precision, recall, and $F_{1}$ score. The $F_{1}$ score is the harmonic average of the precision and recall indicators. The calculation of various indicators is introduced below.

The accuracy is one of the important evaluation metrics to measure the fault diagnosis model. The calculation formula is as follows:

(13) $$\mathrm{accuracy}\left(y,\hat{y}\right)=\frac{1}{n_{\mathrm{samples}\;}}\sum_{i=0}^{n_{\mathrm{samples}\;}-1}I\left({\hat{y}}_{i}=y_{i}\right)\times 100\%$$

where $n_{\mathrm{samples}}$ is the number of all fault samples. *y* is the sample true fault type label. $\hat{y}$ is the predicted fault type label for all samples. I(•) is an indicator function, when *y* is completely equal to $\hat{y}$, the value of I(•) is 1, otherwise the value of I(•) is 0.

For each sample under each fault type, the precision is the ratio of the number of correctly predicted labels to the number of labels predicted to be correct by the classifier. The calculation formula is as follows:

(14) $$\mathrm{precision}\left(y_{s},{\hat{y}}_{s}\right)=\frac{\left|y_{s}\cap {\hat{y}}_{s}\right|}{\left|{\hat{y}}_{s}\right|}\times 100\%$$

where $y_{s}$ is the label data of the real fault type. ${\hat{y}}_{s}$ is the label data for the predicted fault type.

For each sample under each fault type, the recall is the proportion of the number of correct predicted labels in the total number of correct labels. The calculation formula is as follows:

(15) $$\mathrm{recall}\left(y_{s},{\hat{y}}_{s}\right)=\frac{\left|y_{s}⋂{\hat{y}}_{s}\right|}{\left|y_{s}\right|}\times 100\%$$

where $y_{s}$ is the label data of the real fault type. ${\hat{y}}_{s}$ is the label data for the predicted fault type.

The $F_{1}$ score takes into account the precision and recall rate of the classification model at the same time. It is a harmonic average of the precision rate and recall rate of the model. The calculation formula is as follows:

(16) $$F_{1}=2\cdot \frac{\;\mathrm{precision}\;\cdot \;\mathrm{recall}\;}{\;\mathrm{presion}\;+\;\mathrm{recall}\;}$$

### 4.2. Simulation Setup and Data Acquisition

In this paper, the maneuver control instructions provided to the quadrotor UAV are shown in Equations (17)–(19). The attitude maneuver of the quadrotor UAV is carried out according to Equation (1) to Equation (6) and maneuver control instructions. When maneuvering, a fault is injected into the actuator of the quadrotor UAV according to Equations (7) and (8), that is, $t_{f}=t_{d}$. Then, we obtain the residual fault data according to Equation (12). In Equation (12), $\omega_{b}=100$, $b_{0}=13.9237$. The mean value of measurement noise of angular acceleration measured by the sensor is 0, and the variance is 0.1rad/s.

(17) $$\psi =0^{\circ }$$

(18) $$\theta =0^{\circ }$$

(19) $$\varphi =\left\{\begin{array}{cc} 0 & 0\leq t<t_{d}\\ \varphi_{d} & t_{d}\leq t<T \end{array}\right.$$

where ψ is the yaw angle, θ is the pitch angle, φ is the roll angle. $\varphi_{d}$ is the desired roll angle command for maneuvering at $t=t_{d}$.

**Figure 6:**
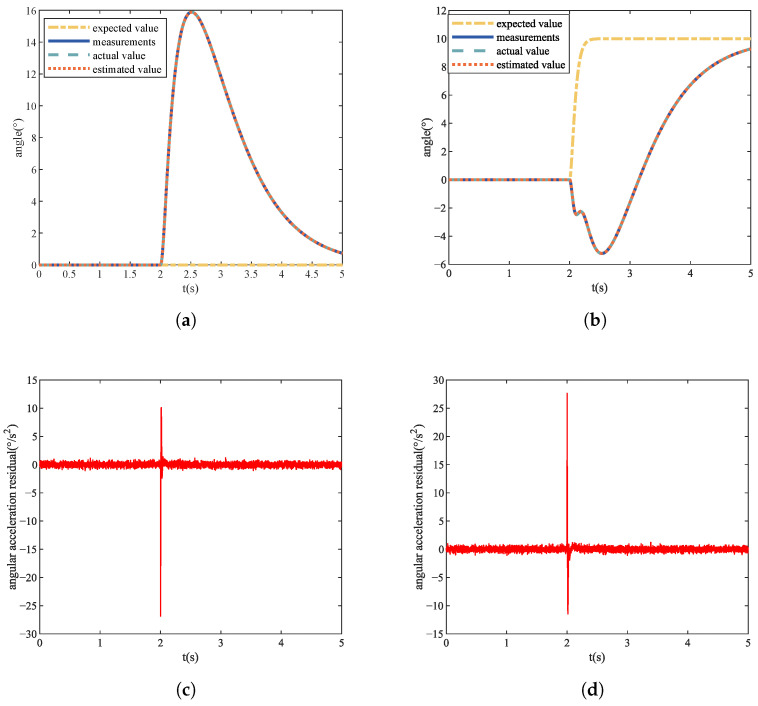
No. 1 actuator constant deviation fault. (**a**) Pitch angle signal value. (**b**) Roll angle signal value. (**c**) Pitch angular acceleration residual signal. (**d**) Roll angular acceleration residual signal.

**Figure 7:**
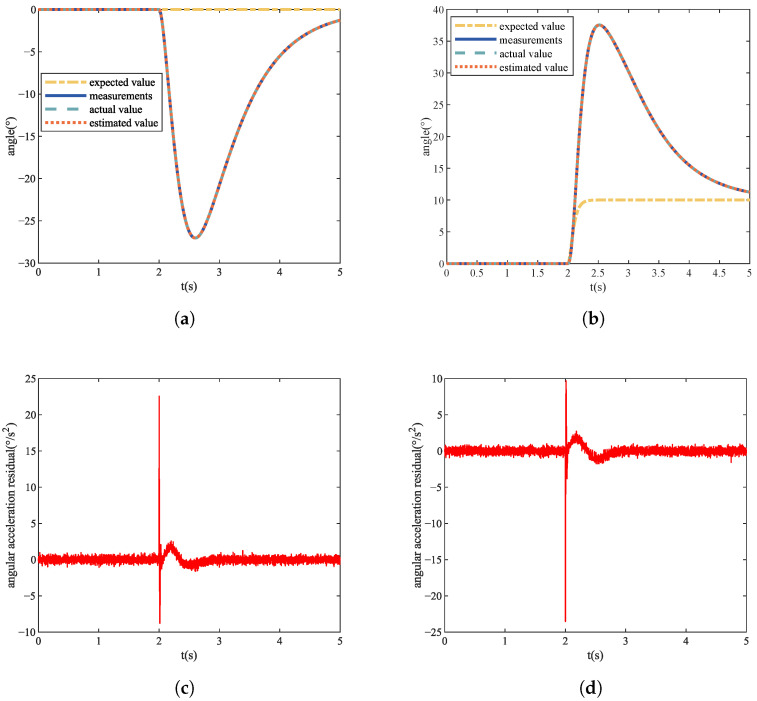
No. 1 actuator gain loss fault. (**a**) Pitch angle signal value. (**b**) Roll angle signal value. (**c**) Pitch angular acceleration residual signal. (**d**) Roll angular acceleration residual signal.

During the experiment, the size of the roll angle maneuver control command $\varphi_{d}$ sent to the quadrotor UAV at $t=t_{d}=2$ s is a random number between $\left[0^{\circ },20^{\circ }\right]$. When $t_{f}=t_{d}=2$ s, the speed constant deviation fault and the speed gain loss fault are injected into the four actuators, respectively. The size of the speed deviation value Δ is a random number between $\left[50\;n/s,800\;n/s\right]$. The angle signal and residual signal of No. 1 actuator with Δ=200n/s fault at $\varphi_{d}=10^{\circ }$ are shown in Figure 6. The size of the speed gain loss factor ρ is a random number between $\left[0.5,1.0\right]$. The angle signal and residual signal of No. 1 actuator with ρ=0.7 fault at $\varphi_{d}=10^{\circ }$ are shown in Figure 7. Each actuator repeats the experiment to obtain 100 sets of data under no-fault conditions, and repeats the experiment to obtain 400 sets of data under the condition of constant speed deviation fault and speed gain loss fault, for a total of 900 sets of data. Finally, we obtain 3600 sets of training data and test data, respectively.

### 4.3. Actuator Fault Localization Analysis

The actuators of the quadrotor UAV are evenly distributed on the fuselage, as shown in Figure 1. When different actuators fail, the state changes of pitch and roll of the quadrotor UAV are different. It can be seen from Figure 6 that when Actuator 1 has a constant rotational speed deviation fault, the pitch state will increase while the roll state will decrease. As can be seen from Figure 7, when Actuator 1 has a failure of rotational speed gain loss, the state of pitch will decrease and the state of roll will increase. Through simulation experiments, we obtained the state change trends of pitch and roll of different actuators when different faults occurred, as shown in Table 1.

**Figure 8:**
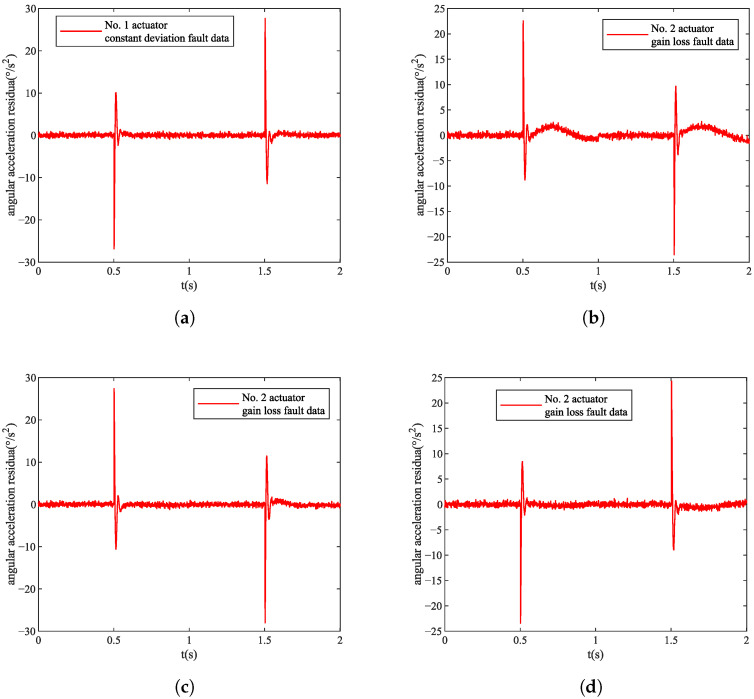
Actuator fault data. (**a**) No. 1 actuator constant deviation fault data. (**b**) No. 1 actuator gain loss fault data. (**c**) No. 2 actuator constant deviation fault data. (**d**) No. 2 actuator gain loss fault data.

It can be seen from Table 1 that when the same fault occurs in different actuators, the combination of angular acceleration residual changes in pitch and roll is different. Therefore, our designed actuator residual feature analysis method can embed the fault localization information into the fault data. In this paper, we set the parameter Δt=1 s of the actuator residual feature analysis method. We obtain the final fault data using a combination of the angular acceleration residuals for pitch and roll using the actuator characterization method; however, when the fault types of the actuators are not unique, different types of faults in different actuators may show the same variation combination of pitch and roll, as shown in Figure 8. The fault data of the constant deviation fault of Actuator 1 and the fault data of the gain loss of Actuator 2 are so similar that it is difficult for the human eye to identify effectively. In order to complete the diagnosis of the fault type of the actuator and the identification of the fault localization at the same time, it is necessary to inject the fault data into the DF model for training and testing.

### 4.4. Comparative Analysis of Fault Diagnosis and Localization

We complete the fault diagnosis and localization of the quadrotor UAV actuator under the condition of unknown fault size by using the DF model to process the fault data. In order to verify the effectiveness of the DF algorithm, we introduce Random Forest (RF) for comparison. The reason we chose RF for comparison is that RF fault diagnosis is widely used and DF is an improved algorithm based on RF. In this paper, the parameters of DF and RF all use default parameters. We completed a final comparative experiment by comparing the two methods and whether ESO was used, as shown in Table 2.

The results of the training and testing accuracy of the above four schemes for the fault diagnosis and localization of the quadrotor UAV actuator are shown in Table 3. It can be seen from Table 3 that the accuracy of the DF scheme is higher than that of the RF scheme.

**Figure 9:**
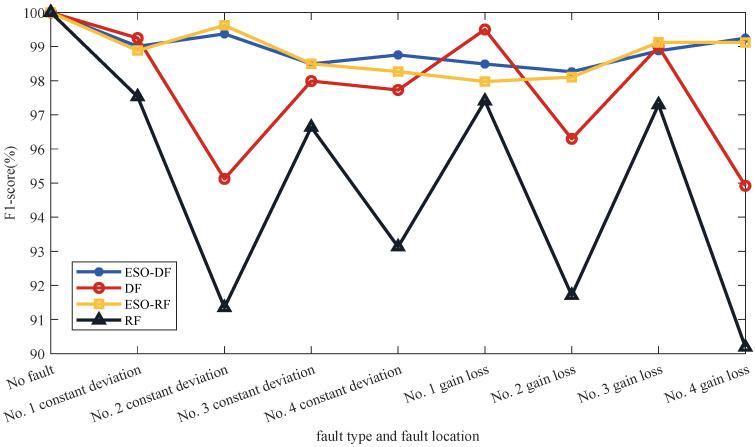
Fault diagnosis and fault localization $F_{1}$ scores of different schemes.

Table 4 shows the accuracy and recall results of four schemes calculated according to Equations (14) and (15) for fault diagnosis and localization of quadrotor UAVs. The results of the $F_{1}$ score calculated by the four schemes according to Equation (16) are shown in Figure 9. As can be seen from the figure, the $F_{1}$ scores of ESO-DF and ESO-RF are relatively close. However, the $F_{1}$ score of ESO-DF has a smaller variation range and is more stable.

According to the fault diagnosis results of various indicators above, we verify that the DF scheme is superior to the RF scheme. Because ESO can better extract the fault features of the system, the effects of fault diagnosis and localization are improved after the introduction of ESO. Through our proposed ESO-DF method, the fault type diagnosis and fault localization identification of the quadrotor UAV actuator under the condition of unknown fault size can be completed at the same time.

## 5. Conclusions

In this paper, we completed the fault diagnosis and localization of unknown fault size of quadrotor UAV actuator by using the ESO-DF based scheme. The scheme achieves high-accuracy fault diagnosis and localization results by combining model-based method with data-driven method. In addition, we designed a residual characteristic analysis method by considering both pitch and roll. This method is straightforward in design, but it can embed fault localization information into fault data. The simulation results indicate that the fault type and fault localization of the actuator can be provided precisely. By comparing the results of other methods, this scheme has better generalization ability and accuracy. In future work, we will try to conduct experimental verification on a real quadrotor UAV.

## Figures and Tables

**Table 1:** The trend of each signal value change when the actuator fails. ↑ means the corresponding value increases, ↓ means the corresponding value decreases.

Actuator Number and Fault	Pitch Angle	Residual Ofpitch Angular Acceleration	Roll Angle	Residual of Roll Angular Acceleration
**No. 1 constant deviation**	↑	↑	↓	↓
**No. 2 constant deviation**	↓	↓	↑	↑
**No. 3 constant deviation**	↓	↓	↓	↓
**No. 4 constant deviation**	↑	↑	↑	↑
**No. 1 gain loss**	↓	↓	↑	↑
**No. 2 gain loss**	↑	↑	↓	↓
**No. 3 gain loss**	↑	↑	↑	↑
**No. 4 gain loss**	↓	↓	↓	↓

**Table 2:** The scheme of actuator fault diagnosis and fault localization.

Number	Specific Scheme
**scheme 1**	ESO-DF
**scheme 2**	DF
**scheme 3**	ESO-RF
**scheme 4**	RF

**Table 3:** Fault diagnosis and fault localization accuracy of different schemes.

Scheme	The Training Accuracy	The Testing Accuracy
**ESO-DF**	99.3000%	99.0500%
**DF**	97.9750%	97.9750%
**ESO-RF**	98.9500%	98.9500%
**RF**	95.5500%	95.5500%

**Table 4:** Fault diagnosis and fault localization precision of different schemes.

Fault Type and Location	Precision				Recall			
	ESO-DF	DF	ESO-RF	RF	ESO-DF	DF	ESO-RF	RF
**No fault**	100.0000%	100.0000%	100.0000%	100.0000%	100.0000%	100.0000%	100.0000%	100.0000%
**No. 1 constant** **deviation**	98.2759%	99.2500%	98.0344%	96.3415%	99.7500%	99.2500%	99.7500%	98.7500%
**No. 2 constant** **deviation**	99.7481%	92.8571%	100.0000%	89.0736%	99.0000%	97.5000%	99.2500%	93.7500%
**No. 3 constant** **deviation**	98.9899%	98.4848%	98.7437%	96.2779%	98.0000%	97.5000%	98.2500%	97.0000%
**No. 4 constant** **deviation**	98.2673%	98.7245%	97.3039%	94.8187%	99.2500%	96.7500%	99.2500%	91.5000%
**No. 1 gain loss**	99.2386%	99.2537%	99.2308%	96.3325%	97.7500%	99.7500%	96.7500%	98.5000%
**No. 2 gain loss**	97.5369%	98.4334%	97.2906%	95.1613%	99.0000%	94.2500%	98.7500%	88.5000%
**No. 3 gain loss**	98.7531%	99.0000%	99.2481%	95.6522%	99.0000%	99.0000%	99.0000%	99.0000%
**No. 4 gain loss**	99.7475%	94.1032%	99.7468%	91.9481%	98.7500%	95.7500%	98.5000%	88.5000%

## Data Availability

Not applicable.

## References

[1] Ai S., Song J., Cai G. (2022). Sequence-to-Sequence Remaining Useful Life Prediction of the Highly Maneuverable Unmanned Aerial Vehicle: A Multilevel Fusion Transformer Network Solution. Mathematics.

[2] D’Amato E., Nardi V.A., Notaro I., Scordamaglia V. (2021). A particle filtering approach for fault detection and isolation of UAV IMU sensors: Design, implementation and sensitivity analysis. Sensors.

[3] Zhang X., Zhao Z., Wang Z., Wang X. (2021). Fault detection and identification method for quadcopter based on airframe vibration signals. Sensors.

[4] Okada K.F.Á., de Morais A.S., Oliveira-Lopes L.C., Ribeiro L. Neuroadaptive Observer-Based Fault-Diagnosis and Fault-Tolerant Control for Quadrotor UAV. Proceedings of the 2021 14th IEEE International Conference on Industry Applications (INDUSCON).

[5] Zhang H., Gao Q., Pan F. An Online Fault Diagnosis Method For Actuators Of Quadrotor UAV With Novel Configuration Based On IMM. Proceedings of the 2020 Chinese Automation Congress (CAC).

[6] Chen Y., Wang B., Liu W., Liu D. On-line and non-invasive anomaly detection system for unmanned aerial vehicle. Proceedings of the 2017 Prognostics and System Health Management Conference (PHM-Harbin).

[7] Tousi M., Khorasani K. Robust observer-based fault diagnosis for an unmanned aerial vehicle. Proceedings of the 2011 IEEE International Systems Conference.

[8] Caliskan F., Hajiyev C. (2016). Active fault-tolerant control of UAV dynamics against sensor-actuator failures. J. Aerosp. Eng..

[9] Fu J., Sun C., Yu Z., Liu L. A hybrid CNN-LSTM model based actuator fault diagnosis for six-rotor UAVs. Proceedings of the 2019 Chinese Control and Decision Conference (CCDC).

[10] Ren X. (2020). Observer design for actuator failure of a quadrotor. IEEE Access.

[11] Gao J., Zhang Q., Chen J. (2020). EKF-based actuator fault detection and diagnosis method for tilt-rotor unmanned aerial vehicles. Math. Probl. Eng..

[12] Ai S., Song J., Cai G. (2021). Diagnosis of sensor faults in hypersonic vehicles using wavelet packet translation based support vector regressive classifier. IEEE Trans. Reliab..

[13] Balestrieri E., Daponte P., De Vito L., Picariello F., Tudosa I. (2021). Sensors and measurements for UAV safety: An overview. Sensors.

[14] Park J., Chang D. Data-driven fault detection and isolation of system with only state measurements and control inputs using neural networks. Proceedings of the 2021 21st International Conference on Control, Automation and Systems (ICCAS).

[15] Song J., Su J., Hu Y., Zhao M., Gao K. (2022). Stability and performance comparison analysis for linear active disturbance rejection control—Based system. Trans. Inst. Meas. Control.

[16] Tang P., Lin D., Zheng D., Fan S., Ye J. (2020). Observer based finite-time fault tolerant quadrotor attitude control with actuator faults. Aerosp. Sci. Technol..

[17] Li X., Chao H., Wang J., Xu Q., Yang K., Mao D. (2021). An Iterative learning extended-state observer-based fuzzy fault-tolerant control approach for AUVs. Mar. Technol. Soc. J..

[18] Zhou Z., Feng J. (2019). Deep forest. Natl. Sci. Rev..

[19] Qin X., Xu D., Dong X., Cui X., Zhang S. (2021). The fault diagnosis of rolling bearing based on improved deep forest. Shock Vib..

[20] Ai S., Shang W., Song J., Cai G. Fault Diagnosis of the Four-Rotor Unmanned Aerial Vehicle using the Optimized Deep Forest Algorithm based on the Wavelet Packet Translation. Proceedings of the 2021 8th International Conference on Dependable Systems and Their Applications (DSA).

[21] Zhao K., Song J., Wang X. (2021). ESO-KELM-based minor sensor fault identification. J. China Univ. Posts Telecommun..

[22] Guzmán-Rabasa J.A., López-Estrada F.R., González-Contreras B.M., Valencia-Palomo G., Chadli M., Perez-Patricio M. (2019). Actuator fault detection and isolation on a quadrotor unmanned aerial vehicle modeled as a linear parameter-varying system. Meas. Control.

[23] Du B., Polyakov A., Zheng G., Quan Q. (2019). Quadrotor trajectory tracking by using fixed-time differentiator. Int. J. Control.

[24] Yu B., Zhang Y., Qu Y. MPC-based FTC with FDD against actuator faults of UAVs. Proceedings of the 2015 15th International Conference on Control, Automation and Systems (ICCAS).

[25] Zhao Z., Guo B. (2017). A novel extended state observer for output tracking of MIMO systems with mismatched uncertainty. IEEE Trans. Autom. Control.

